# Effect of Molecular Weight and Chemical Structure of Terminal Groups on the Properties of Porous Hollow Fiber Polysulfone Membranes

**DOI:** 10.3390/membranes13040412

**Published:** 2023-04-05

**Authors:** Dmitry Matveev, Alisa Raeva, Ilya Borisov, Vladimir Vasilevsky, Yulia Matveeva, Azamat Zhansitov, Svetlana Khashirova, Vladimir Volkov

**Affiliations:** 1 A.V. Topchiev Institute of Petrochemical Synthesis RAS, 29 Leninsky Prospekt, 119991 Moscow, Russia; 2 Progressive Materials and Additive Technologies Center, Kabardino-Balkarian State University Named after H.M. Berbekov, St. Chernyshevsky, 173, 360004 Nalchik, Russia

**Keywords:** polysulfone, synthesis, molecular weight, chemical structure, terminal groups, hollow fiber membranes, porous support, gas permeability

## Abstract

For the first time, polysulfones (PSFs) were synthesized with chlorine and hydroxyl terminal groups and studied for the task of producing porous hollow fiber membranes. The synthesis was carried out in dimethylacetamide (DMAc) at various excesses of 2,2-bis(4-hydroxyphenyl)propane (Bisphenol A) and 4,4′-dichlorodiphenylsulfone, as well as at an equimolar ratio of monomers in various aprotic solvents. The synthesized polymers were studied by nuclear magnetic resonance (NMR), differential scanning calorimetry, gel permeation chromatography (GPC), and the coagulation values of 2 wt.% PSF polymer solutions in *N*-methyl-2-pyrollidone were determined. According to GPC data, PSFs were obtained in a wide range of molecular weights M_w_ from 22 to 128 kg/mol. NMR analysis confirmed the presence of terminal groups of a certain type in accordance with the use of the corresponding monomer excess in the synthesis process. Based on the obtained results on the dynamic viscosity of dope solutions, promising samples of the synthesized PSF were selected to produce porous hollow fiber membranes. The selected polymers had predominantly –OH terminal groups and their molecular weight was in the range of 55–79 kg/mol. It was found that porous hollow fiber membrane from PSF with M_w_ 65 kg/mol (synthesized in DMAc with an excess of Bisphenol A 1%) has a high helium permeability of 45 m^3^/m^2^∙h∙bar and selectivity α (He/N_2_) = 2.3. This membrane is a good candidate to be used as a porous support for thin-film composite hollow fiber membrane fabrication.

## 1. Introduction

Polysulfone (PSF) is one of the most common synthetic polymeric materials used to produce membranes for various purposes. PSF membranes are used in the processes of micro- and ultrafiltration (UF), hemodialysis, pervaporation, gas separation, etc. [1,2,3]. Such a wide application areas is due to its availability, chemical inertness, thermal stability, high mechanical strength, and the possibility of simple modification [4]. In addition, hollow fiber PSF membranes are actively used as a support for the producing of thin-film composite membranes [5].

Most of the currently existing studies in the field of membrane application of PSFs are based on the use of its commercially available brands (the manufacturers: BASF, Solvay, Sigma-Aldrich, etc.), as well as their adaptation by chemical modification of the polymer or the hybrid materials creation [2,3]. In particular, the functionalization of PSF is a common method for changing its chemical and physical properties for the subsequent production of membranes for targeted applications (Table 1). From Table 1, it can be seen that in the majority of cases, chemical modification is carried out on the original commercial PSF. At the same time, there is a slight difference in the molecular masses (MM) of the polymers used: M_n_ = 22–29 kg/mol, M_w_ = 17.6; 35; 58 kg/mol. However, the molecular weight of the polymer is an important parameter that significantly affects the structure and characteristics of the resulting membranes [6,7].

It should be noted that the chemical modification of PSF is carried out for the subsequent production of exclusively flat-type membranes (see Table 1). In the case of a hollow fiber configuration, to improve the properties of membranes obtained from PSF, a common approach is to introduce various additives and fillers into the membrane matrix, i.e., the creation of mixed-matrix membranes (MMMs) [22,23,24,25,26,27,28,29]. To obtain MMMs and unmodified hollow fiber membranes from PSF, as a rule, commercial grades of PSF are used [22,23,24,25,26,27,28,29,30,31,32,33,34]. However, due to the limited choice of molecular weights and chemical composition, the use of industrial polymers may hinder the ability to prepare dope solutions with controlled properties (viscosity, stability upon contact with a coagulant, coagulation rate, etc.). This, in turn, can make it difficult to simultaneously achieve high values of permeability and small pore size for porous hollow fiber membranes in the process of their spinning from a solution in a dry-jet wet process. This issue is important in the case of producing porous supports for high-performance thin-film composite membranes for gas separation. The porous support should provide a high value of gas flux to reduce the mass transfer resistance through the composite membrane and a fine pore size distribution for deposition of a thin defect-free selective layer.

A possible solution to this challenge is not the adaptation of commercial grades of PSF, but the creation and development of methods for the synthesis of PSF that meets all the requirements for membrane polymers. In the framework of this work, for the first time, polysulfones with chlorine and hydroxyl terminal groups were synthesized and studied for the task of porous hollow fiber membranes fabrication. One of the effective approaches to control the molecular weight characteristics of synthesized polymeric materials according to the mechanism of the reaction of bimolecular nucleophilic substitution is synthesis with a variation of its regimes and the nature of the solvent [35]. With this in mind, the method of changing the ratio of monomers according to the rule of non-equivalence of functional groups in the process of polycondensation was used as an effective way to obtain polymeric materials with specified molecular weight characteristics and containing certain terminal groups. It was expected that this would make it possible to determine the synthesis conditions, in particular, the optimal ratio of monomers and comonomers, to obtain the PSF samples with the necessary characteristics for the preparation of fine porous hollow fiber supports for thin-film hollow fiber membrane fabrication. One possible application of the support might be developing composite membranes with selective layers from polydecylmethylsiloxane (PDecMS), which was previously implemented on flat-sheet type membranes [36]. PDecMS demonstrates high separating properties in the removal of C_3+_ hydrocarbons from their mixture with methane [36,37].

## 2. Materials and Methods

### 2.1. Materials

PSF was synthesized using 2,2-bis(4-hydroxyphenyl)propane (Bisphenol A, 97%), N,N-dimethylacetamide (DMAc, η = 0.92 mPa·s (25 °C), chemically pure), and *N*-methyl-2-pyrollidone (NMP, η = 1.66 mPa·s (25 °C), chemically pure) provided by Acros Organics (Geel, Belgium); 4,4′-dichlorodiphenylsulfone (DCDPS, 99%) was purchased from Alfa Aesar (Heysham, UK), potassium carbonate (chemically pure) and dimethyl sulfoxide (DMSO, η = 2.00 mPa·s (25 °C), chemically pure) were purchased from Reachem (Moscow, Russia).

To prepare the dope solutions, NMP was used as a solvent and polyethylene glycol with an average molecular weight of 400 g/mol (PEG-400, Acros Organics (Geel, Belgium)) as a pore-forming additive.

### 2.2. Synthesis of PSF with Different Terminal Groups

**Figure 1:**
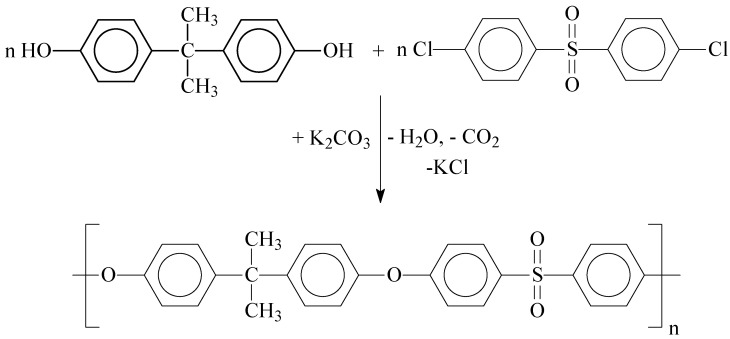
PSF synthesis scheme.

PSF samples were obtained by high-temperature polycondensation by the nucleophilic substitution mechanism in aprotic dipolar solvents at different ratios of Bisphenol A and DCDPS monomers. The synthesis (Figure 1) was carried out in the presence of an alkaline agent, potassium carbonate, to convert the hydroxyl groups of Bisphenol A into active phenolate groups, with constant distillation of water from the reaction zone. The polycondensation was carried out in a glass reactor system equipped with an overhead stirrer, a thermocouple, an inert gas capillary, a Dean–Stark trap, and a reflux condenser. In order to study the effect of terminal active groups of PSF, polymers with –Cl and –OH terminal groups were synthesized. The syntheses were carried out in a DMAc media at molar ratios of Bisphenol A: DCDPS 1:(1.01–1.03) and (1.01–1.025):1, respectively. To determine the optimal solvent, PSF samples were synthesized in various dipolar solvents (DMAc, NMP, and DMSO) at an equimolar ratio of Bisphenol A and DCDPS monomers. The syntheses were carried out at the boiling temperature of the chosen solvent. Subsequently, the reaction solution was cooled to 30 °C and precipitated by spraying into distilled water acidified with hydrochloric acid. The polymer was repeatedly washed with hot distilled water to remove low molecular weight reaction products and the solvent. The polymer powder was dried in a vacuum oven at 120 °C for 12 h. The molecular weight of the synthesized PSF samples was controlled by the ratio of the starting monomers according to the nonequivalence rule. Table 2 presents the obtained PSF samples, as well as the conditions for their synthesis.

### 2.3. Study of Synthesized PSFs

#### 2.3.1. Nuclear Magnetic Resonance (NMR) Method

High-resolution ^1^H NMR spectra were obtained for solutions in CDCl_3_ according to the standard procedure on a Bruker AVANCE III HD 400 NMR spectrometer (Rheinstetten, Germany).

#### 2.3.2. Differential Scanning Calorimetry Method

The analysis was carried out on a Perkin Elmer DSC 4000 (Waltham, MA, USA) instrument in a nitrogen atmosphere in the range from 25 to 250 °C. The scanning rate during heating was 10 °C/min. The glass transition temperature obtained during the second heating of the sample was taken as the result of the analysis.

#### 2.3.3. Gel Permeation Chromatography Method

Gel-permeation chromatography (GPC) analysis of the polymers was performed on a Waters system with a differential refractometer (Waters, Milford, MA, USA) (Chromatopack Microgel-5; eluent, chloroform (99%, AppliChem GmbH, Darmstadt, Germany); flow rate, 1 mL/min). The molecular weights and polydispersity were calculated by a standard procedure relative to monodispersed polystyrene standards.

#### 2.3.4. Determination of Coagulation Values

Coagulation values (CVs, g∙dL^−1^) of distilled water for PSF in NMP were determined as the quantity (g) of water needed to cause the phase separation of 100 mL (1 dL) of 2 wt.% polymer solution by titration method (T = 25 °C). Coagulation was observed visually until the polymer solution did not become homogeneous in 24 h [38]. CVs are expressed as the weight (g) of the non-solvent per 1 dL of 2 wt.% polymer solution.

### 2.4. Dope Solution Preparation

To produce PSF hollow fiber membranes, the dope solution was chosen, which was used by us earlier [39,40,41,42]. PSF and PEG-400 (mass ratio 1:1.36) were placed in a thermostatically controlled reactor and stirred at a speed of 150 rpm at a temperature of 25 °C. NMP was then added to the system, while increasing the stirring speed to 500 rpm. Under these conditions, the solution was mixed until homogeneity was achieved (at least 16 h). The concentration of PSF in the dope solution assumed a value of 21 wt.%. Before hollow fiber membrane spinning, the polymer solution was filtered under a nitrogen pressure of 1.8–2.0 bar through a stainless-steel mesh (cutoff rating 4–5 μm). After the filtration procedure, the polymer solution was degassed under vacuum overnight.

### 2.5. Polymer Solution Viscosity Measurement

To measure the viscosity of dope solutions, a Brookfield DV III-Ultra rotational viscometer (Brookfield Engineering Labs, Middleboro, MA, USA) was used. In addition, the dynamic viscosity was determined for solutions of PSF in NMP and DMAc (polymer concentration 21 wt.%). Measurements for each solution were carried out at a temperature of 22 °C.

### 2.6. Study of the Phase Inversion Kinetics

The phase inversion kinetics of dope solutions were studied by the method of measuring the coagulation rate in a “limited” layer of a polymer solution [43]. This technique makes it possible to simulate the formation of a polymer membrane of a given thickness and to visualize the structure formation in an asymmetric membrane. The phase inversion kinetics were estimated from the coagulation rate υ of the polymer solution layer, which is calculated as the ratio of the total thickness of the polymer layer (μm) to its coagulation time (s). The rate of the coagulation front was averaged over three measurements. Distilled water was used as a coagulant.

### 2.7. Hollow Fiber Membranes Preparation

Samples of PSF hollow fiber membranes were prepared via a dry/wet phase inversion using the setup described elsewhere [44]. To form the lumen of the hollow fiber, distilled water was poured inside. An annular spinneret with outer/inner diameters of 0.8/0.5 mm was used. After spinning, the samples of hollow fiber membranes were sequentially washed with tap water, then with ethanol for 2 h, and then with n-hexane for 2 h, after which they were dried in air in ambient conditions.

### 2.8. Study of PSF Hollow Fiber Membranes

#### 2.8.1. Gas Transport Properties

The pure gas (He, N_2_) permeances through hollow fiber membranes were measured using a volumetric membrane apparatus. The gas flow was introduced to the lumen side of the hollow fiber. The volumetric gas flow passing through the membrane was measured using a Dry Gas Meter (SHINAGAWA, Tokyo, Japan). Gas permeance measurements were carried out at room temperature (23 ± 1 °C) under transmembrane pressure from 0.5 to 2 bar, while the permeate gas pressure was kept constant at 1 bar. Gas permeance was calculated using the equation:

(1) $$\frac{P}{l}=\frac{Q}{p\cdot S},$$

where *Q*—gas volumetric flow rate through the membrane, m^3^/h; *p*—transmembrane pressure, atm; *S*—membrane surface, m^2^. Ideal selectivity was calculated using the equation:

(2) $$\alpha =\frac{\left(P_{1}/l\right)}{\left(P_{2}/l\right)}=\frac{P_{1}}{P_{2}},$$

where (*P_1_/l*)—He permeance, m^3^/(m^2^·h·atm); (*P_2_/l*)—N_2_ permeance, m^3^/(m^2^·h·atm).

#### 2.8.2. Porosimetry

The porosity of PSF hollow fiber membranes was determined using the instrument POROLIQ 1000 ML (POROMETER, Nazareth, Belgium). Membrane pore size analysis was performed by a liquid–liquid displacement method using water-saturated isobutanol and isobutanol-saturated water as a solvent pair. The porous structure was characterized by the diameter of the largest pore (d_max_) and the diameter of the smallest pore (d_min_), as well as the mean flow pore size d_MFP_ (MFP). The MFP value is defined as the pore size at which 50% of the flux penetrates through the larger pores and 50% of the flux penetrates through the smaller pores of the membrane skin layer.

#### 2.8.3. Scanning Electron Microscopy

The membrane structure was studied by scanning electron microscopy (SEM) using a Hitachi «Tabletop TM 3030 Plus» microscope with a high-sensitivity low-vacuum secondary electron detector (Hitachi High Technologies Corporation, Tokyo, Japan). The accelerating voltage during image acquisition was 15 kV. The thickness of the gold layer was 5 nm.

## 3. Results and Discussion

### 3.1. Properties of Synthesized PSFs

**Figure 2:**
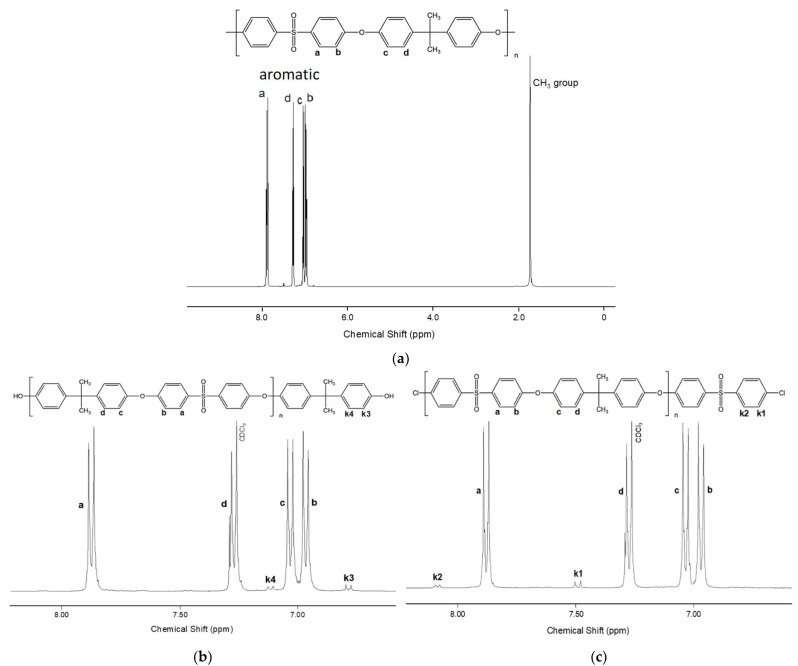
NMR spectra of the synthesized PSF with different terminal groups: (**a**) general view of the PSF spectrum, (**b**) –OH groups, (**c**) –Cl groups.

The synthesized polymers were characterized by ^1^H NMR spectroscopy. Each PSF sample was dissolved in CDCl_3_. Peaks at chemical shift values δ7.88 H-a (4H, d), δ7.28 H-d (4H, d), δ7.03 H-c (4H, d) and δ6.97 H-b (4H, d) (Figure 2a–c) were assigned to hydrogen protons in the aromatic rings of the main polymer chain. The singlet peak at δ1.73 (6H, s) (Figure 2a) corresponds to the hydrogen protons of the methyl groups of the main chain. Signals k1 δ7.49 (2H, d) and k2 δ8.08 (2H, d) (Figure 2b) were assigned to protons of terminal aromatic rings connected to chlorine terminal groups, and signals of protons k3 δ6.77 (2H, d) and k4 δ7.11 (2H, d) (Figure 2c)—to the protons of aromatic rings connected to hydroxyl terminal groups.

Based on the spectral analysis, the ratio of –OH and –Cl terminal groups obtained in the synthesis process was calculated (Table 3). This ratio was determined by comparing the integral areas of the received signals k3 and k1, which refer to the protons of the terminal aromatic rings connected to the hydroxyl and chlorine terminal groups, respectively. From Table 3, a regular correlation can be noticed: the proportion of terminal groups of a certain type increases with an increase in the excess of the corresponding monomer. It should be noted that at an equimolar ratio of monomers, the proportion of terminal –OH groups prevails, regardless of the solvent used (1.2–1.3 times). The length of the polymer chain was also calculated by comparing the normalized (per one proton) integral areas of the signals of the inner and terminal groups. The calculated number of links in the polymer chain is shown in Table 3.

The study of the molecular weight characteristics of the synthesized polymers by GPC showed that the synthesis of PSF with terminal group blocking resulted in the production of polymers with a unimodal molecular weight distribution. At the same time, as can be seen from Table 3, with an increase in the excess of DCDPS or Bisphenol A, the molecular weight of the polymer decreases. Thus, in the case of an increase in the excess of DCDPS from 1 to 3 %, the M_w_ of the synthesized PSF decreases from 65 to 57 kg/mol, and with an increase in the excess of Bisphenol A from 1 to 2.5 %, the M_w_ decreases from 128 to 37 kg/mol. The stoppage of the polymer chain growth in nonequilibrium polycondensation, when it is carried out in the presence of an excess of one of the initial substances, is probably caused by the fact that at a certain stage of the reaction the resulting macromolecules will have the same functional groups of the excess component at both ends of the chain, excluding further elementary reactions leading to polymer chain growth [45]. The decrease in the molecular weight of the polymers with an increase in the excess of both monomers is confirmed both by GPC and by NMR estimates. Moreover, the two methods demonstrate good convergence (see Table 3).

In addition, it is worth noting the difference in the molecular weights of polymers synthesized at an equimolar ratio of monomers but in different solvents. PSF with the highest molecular weight (M_w_ = 79 kg/mol) is obtained by synthesis in NMP, with the lowest (M_w_ = 23 kg/mol)—in DMSO. This is apparently due to the influence of the nature of the solvent on the processes of structuring. DMSO exhibits a higher solvating capacity than other aprotic dipolar solvents. The high solvating ability of DMSO with respect to potassium phenolates leads to an increase in their nucleophilicity and, consequently, to an increase in the rate constant of the reaction leading to the formation of a gel fraction, which, apparently, is associated with the occurrence side branching reactions together with the main reaction of obtaining linear PSF. At the same time, the presence of even trace amounts up to 0.5 % DMSO leads to undesirable structuring processes during polymer processing, because DMSO acts as a radical initiator of destruction [46,47,48]. The content of trace amounts (0.05 wt.%) of DMSO in the polymer can lead to an increase in the viscosity of the melt and darkening of the polymer [46], which is associated with a strong solvation of the terminal reaction centers of the resulting polymers by DMSO; this makes it difficult to completely remove it from the polymer.

**Figure 3:**
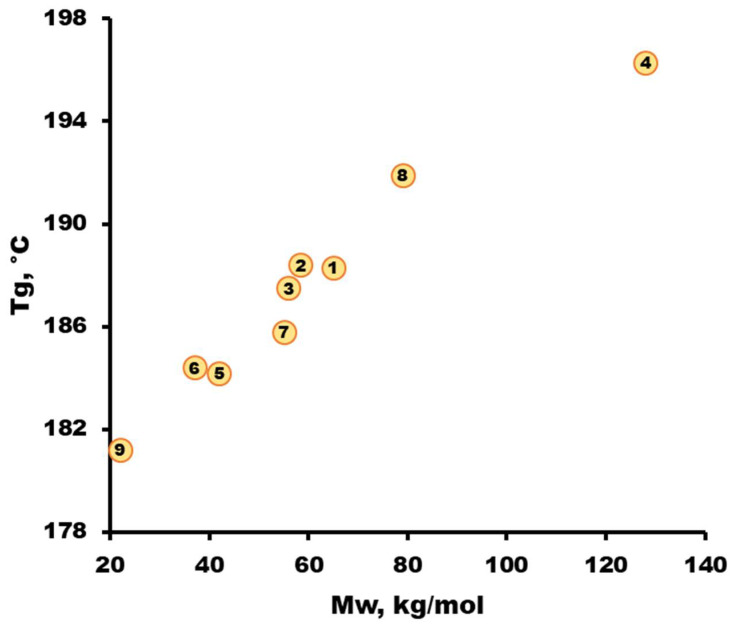
Dependence of the glass transition temperature determined by DSC on the molecular weight of the synthesized PSF. The sample numbers are the same as those in Table 3.

The glass transition temperature T_g_ of the synthesized polymers was determined by DSC. The data obtained are presented in Figure 3. From Figure 3, it can be observed that with an increase in the molecular weight of the polymer M_w_, a regular increase in its glass transition temperature is observed, regardless of the PSF synthesis conditions. Similar correlations are observed for commercial grades of PSF. In [49] for PSF grades Ultrason S 2010 (M_w_ = 53.8 kg/mol) and Udel 1700 (M_w_ = 34.6 kg/mol), the glass transition temperatures T_g_ are 188 and 186 °C, respectively.

**Figure 4:**
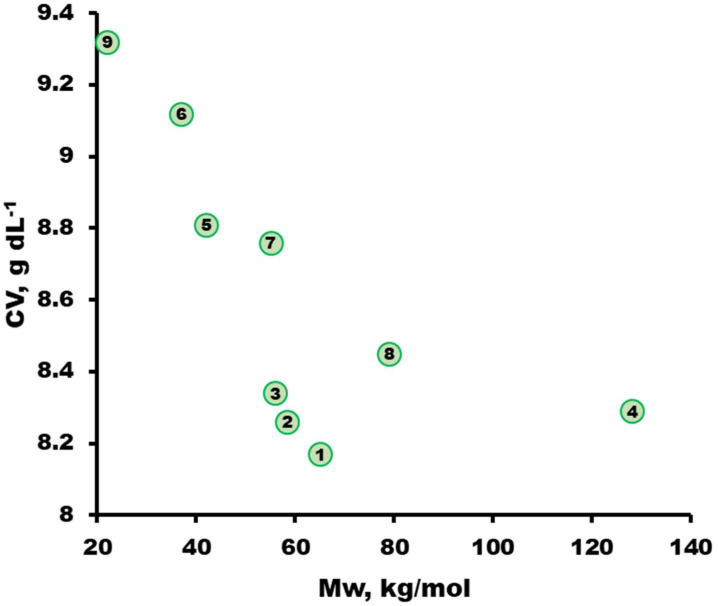
Coagulation values dependence obtained by titration of 2 wt.% polymer solution of synthesized PSF in NMP by water. The sample numbers are the same as those in Table 3.

Coagulation values (CVs) were obtained by titration of a 2 wt.% polymer solution of synthesized PSF samples in NMP with water. The CV was found to be highly dependent on the molecular weight of the polymer (Figure 4): the higher the molecular weight of the polymer, the lower the CV. At the same time, lower CV values are observed for synthesized PSFs with –Cl terminal groups (PSF-1–PSF-3). This means that a smaller amount of water is required for the phase separation of the polymer solution. This circumstance may be due to the lower solubility of the polymers with an increase in their molecular weight, as well as to the presence of chlorine terminal groups.

### 3.2. Properties of Dope Solutions

**Figure 5:**
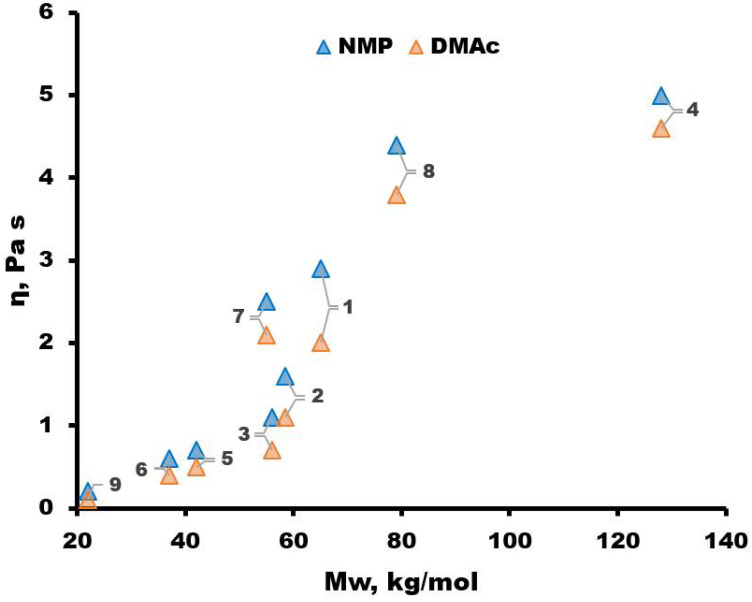
Dependence of the dynamic viscosity of the PSF/NMP and PSF/DMAc solutions on the molecular weight of PSF. The sample numbers are the same as those in Table 3.

The viscosity is an important parameter that largely determines the properties of the hollow fiber membranes [50]. For this reason, solutions of the synthesized PSFs in NMP and DMAc with a polymer concentration of 21 wt.% were studied (Figure 5). It should be noted that it was not possible to completely dissolve the polymers in DMF and DMSO, that is, to obtain a homogeneous solution. This may be due to different Hansen solubility parameters. For example, for PSF of the Ultrason S 6010 brand, the series of solvents as their “affinity” to the polymer deteriorates is as follows: NMP-DMAc-DMF-DMSO [51,52]. It can be seen from Figure 5 that with an increase in the molecular weight of the synthesized PSFs, the dynamic viscosity of 21 wt.% solutions increases from 0.3 to 5.1 Pa·s in the case of the NMP solvent and from 0.2 to 4.6 Pa·s in the case of DMAc. At the same time, for polymer solutions with DMAc solvent, the value of dynamic viscosity is less than with NMP, by a factor of 1.09–1.57, depending on the molecular weight of PSF. It is worth highlighting the polymers PSF-7 (predominant -OH terminal groups) and PSF-2 and PSF-3 (predominant -Cl terminal groups) with similar molecular weights M_w_ = 55–58 kg/mol. The polymer solution with PSF-7 in NMP demonstrates a viscosity of 2.5 Pa·s, PSF-2 and PSF-3—1.7 and 1.1 Pa·s, respectively. This circumstance may be associated with different solubility depending on the terminal groups of the synthesized polymers.

**Figure 6:**
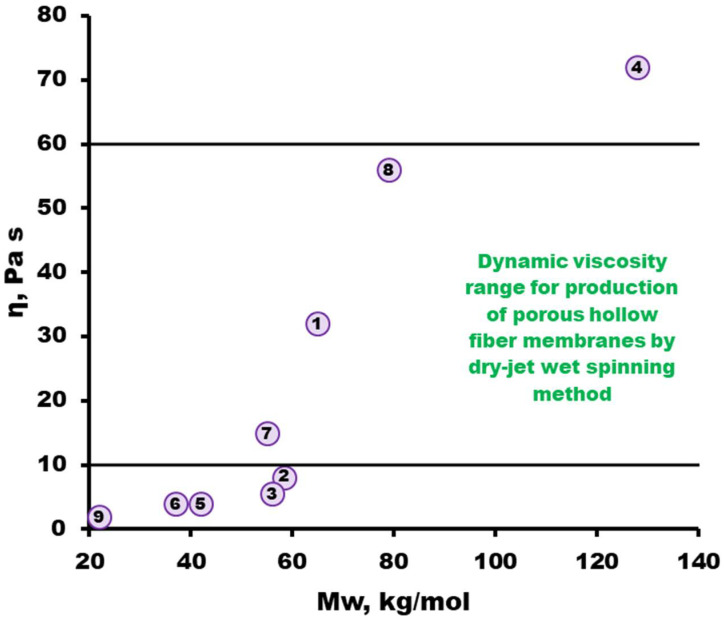
Dependence of the dynamic viscosity of the PSF/NMP/PEG-400 (21/49/30 wt.%) dope solutions on the molecular weight of the polymer. The sample numbers are the same as those in Table 3.

To increase the porosity of membranes, pore-forming additives, in particular PEG, were added to the dope solution [53]. Figure 6 shows the viscosity of PSF/NMP/PEG-400 dope solutions (21/49/30 wt.%). From Figure 6, it can be seen that the addition of PEG leads to a significant increase in the viscosity of the dope solutions. The viscosity of dope solutions used to produce hollow fiber membranes is typically in the range of 10–60 Pa·s. In the case of dynamic viscosities below this range, it is rather difficult to obtain defect-free hollow fiber membranes by the dry-jet wet spinning method. Figure 6 shows that the dope solutions of the synthesized PSF-1, PSF-7 and PSF-8 samples of the selected composition meet the viscosity requirements for spinning hollow fiber membranes. For this reason, dope solutions with these polymers were selected for further study. The molecular weight of polymers PSF-1, PSF-7 and PSF-8 is in the range of 55–79 kg/mol. It is also worth noting that the proportion of –OH terminal groups prevails in the selected polymers (see Table 3).

Furthermore, the coagulation kinetics of dope solutions were studied for PSF-1, PSF-7 and PSF-8. The measurements were carried out in a “limited” layer [43] 300 µm thick at a temperature of 21 °C. The choice of distilled water as a coagulant is due to the fact that it was used as a bore fluid in the process of hollow fibers spinning. The phase inversion kinetics were estimated from the rate of the passage of the deposition front over the entire thickness of the polymer solution layer. At the end of the movement of this front, only the primary polymer matrix is formed. Clearly, this is followed by the removal of the residual solvent and the final coagulation of the polymer solution. Table 4 shows the results of determining the coagulation rate υ for the dope solutions based on the three selected PSF samples for the further production of hollow fibers. It can be seen from Table 4 that the coagulation rate changes from 3.9 to 6 μm/s with an increase in the molecular weight of the synthesized polymer and the dope solution viscosity. A similar effect was found in [50], where the viscosity of the dope solution was controlled by changing its temperature. An increase in viscosity can limit the mutual penetration of solvent molecules (NMP) and non-solvent (distilled water) in opposite directions from the polymer solution and into it.

### 3.3. Study of Porous Hollow Fiber Membranes

**Figure 7:**
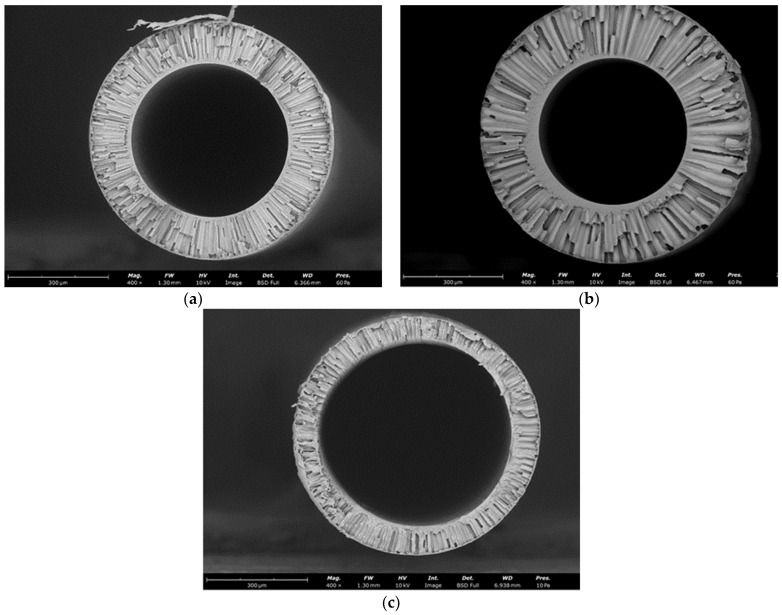
SEM micrographs of the cross-section of hollow fiber membranes obtained from synthesized (**a**) PSF-1, (**b**) PSF-7 and (**c**) PSF-8.

Based on the viscosity data obtained, porous hollow fiber membranes were produced from PSF/NMP/PEG-400 dope solutions with PSF-1, PSF-7, and PSF-8 samples using the dry-jet wet spinning method. Figure 7 shows SEM micrographs of the cross-section of the formed membranes. It can be seen that the obtained hollow fiber membranes have an asymmetric structure with a thin selective layer and a porous supporting layer penetrated by finger-shaped macrovoids. It can further be noted that there were no visible changes in the morphology of the hollow fiber membranes for various PSF samples.

From the data of SEM micrographs, the geometric parameters of hollow fiber membranes were estimated: average outer D_out_ and inner diameters, fiber wall thickness δ. The results obtained are shown in Table 5. Significant differences in the geometry of the resulting hollow fiber membranes can be seen. The smallest wall thickness δ of 100 µm has a hollow fiber membrane obtained from a PSF-8 sample (M_w_ = 79 kg/mol). The outer diameter of this membrane is 0.8 mm. The largest outer diameter of 0.9 mm and wall thickness of 200 µm are demonstrated by the membrane from the PSF-7 sample (M_w_ = 55 kg/mol). The results obtained can be explained by the different viscosities of the dope solutions (Table 4).

The gas transport properties of hollow fiber membranes were studied by the volumetric method for individual gases: helium, nitrogen. Differences in the molecular weights of these gases make it possible to estimate the contribution of the Knudsen flow regime by the values of ideal selectivities, that is, the ratios of the permeability coefficients for individual gases. The obtained gas permeability and selectivity data are presented in Table 5. The hollow fiber membrane prepared from PSF-1 (M_w_ = 65 kg/mol) exhibits a He permeability of 45 m^3^/bar·m^2^·h and a He/N_2_ gas selectivity of 2.3. This value of ideal selectivity indicates that gas transport in the resulting membrane is close to the Knudsen regime. Thus, in the regime of the Knudsen flow of gases, α for a pair of gases He/N_2_ is 2.65. Hollow fiber membranes obtained from the synthesized polymer with a molecular weight of 55 kg/mol (PSF-7) demonstrate high permeability values: P/l (He) = 73 m^3^/bar·m^2^·h, but lower selectivity: α (He/N_2_) = 1.6. The PSF-8 hollow fiber membrane with the highest molecular weight (M_w_ = 79 kg/mol) demonstrates both the lowest gas permeability values (P/l (He) = 2.3 (m^3^/bar·m^2^·h)) and low selectivity values (α (He/N_2_) = 1.9). The selectivity values for hollow fiber membranes obtained from the synthesized PSF-7 and PSF-8 polymers indicate a mixed gas flow regime, average between the Knudsen and Poiseuille flows (α = 1.0 for the He/N_2_ gas pair). The results on gas permeability are not inferior to the results for hollow fiber membranes made from commercial PSF. For example, PSF hollow fiber membranes, fabricated from PSF commercial sample Ultrason S 6010 (BASF), demonstrated helium permeability 15–63 m^3^/bar·m^2^·h and selectivity α (He/CO_2_) 3.28–1.47, depending on the draw ratio [44].

Data on the gas permeability of hollow fiber membranes correlate with the results of measurements of the pore size distribution. As can be seen from Table 5, the largest pore size (d_max_) well correlates with the obtained He/N_2_ ideal selectivity. The highest d_max_ of 81 nm has the membrane PSF-7 with the highest permeance and the lowest selectivity of 1.6. For the hollow fiber membrane obtained from PSF-1 (M_w_ = 65 kg/mol), the d_max_ value is only 28 nm, which resulted in the highest He/N_2_ selectivity of 2.3. For the hollow fiber membrane made of PSF-8, the d_max_ value (35 nm) is in between PSF-1 and PSF-7, which explains the value of its selectivity α (He/N_2_) = 1.9.

## 4. Conclusions

For the first time, PSF samples with various terminal groups (chlorine and hydroxyl) were synthesized in a wide range of molecular weights for the task of producing porous hollow fiber membranes. Synthesis was carried out by high-temperature polycondensation by the mechanism of nucleophilic substitution in DMAc at various excesses of Bisphenol A or DCDPS monomers, as well as at equimolar ratios of monomers in various solvents. Based on NMR analysis, a regular increase in the proportion of terminal groups of a certain type was shown with an increase in the excess of the corresponding monomer. However, at an equimolar ratio of monomers, the proportion of PSF with –OH terminal groups prevails by a factor of 1.2–1.3, depending on the solvent used. It was shown by DSC that an increase in the polymer molecular weight M_w_ is accompanied by an increase in its glass transition temperature, regardless of the PSF synthesis conditions.

From the synthesized polymers, two-component PSF/(NMP, DMAc) and three-component PSF/NMP/PEG-400 dope solutions were prepared. Based on the results of the dynamic viscosity of dope solutions, promising PSF samples were selected to produce porous hollow fiber membranes. In the course of the work, it was found that these samples have a higher proportion of –OH terminal groups and the molecular weight of the polymers is in the range of 55–79 kg/mol. Porous asymmetric hollow fiber membranes were formed from dope solutions with selected three PSF samples (PSF-1, PSF-7, and PSF-8). It was found that hollow fiber membranes from PSF-1 (synthesized in DMAc with an excess of Bisphenol A 1 %, molecular weight of 65 kg/mol) have high He permeability (45 m^3^/bar·m^2^·h) and ideal selectivity He/N_2_ of 2.3. The results obtained make the PSF-1 a good candidate for obtaining porous support and further thin-film composite hollow fiber membrane fabrication.

## Figures and Tables

**Table 1:** Functionalization of PSF to produce membranes for targeted use (literature data).

Functional Group	PSF Supplier	PSF MM, kg/mol	Membrane Configuration	Application	Ref.
2-methoxyethyl acrylate	Sigma-Aldrich, China	M_n_ ~22	Flat-sheet	Hemodialysis	[8]
quaternary ammonium groups N(CH_3_)_3_^+^-CH_2_–	Sigma-Aldrich, Australia	M_w_ ~35	Flat-sheet	Dialysis	[9]
chitosan	Sigma-Aldrich, St. Louis, MO, USA	M_n_ ~22	Flat-sheet	Hemodialysis	[10]
azide groups N_3_-(CH_2_)_3_-N(CH_3_)_2_^+^-CH_2_–	Sigma-Aldrich, China	*M*_w_ ~58	Flat-sheet	Anion exchange membranes	[11]
–SO_3_H groups	Sigma-Aldrich, St. Louis, MO, USA	M_w_ ~35	Flat-sheet	Seawater desalination	[12]
naphthalene moieties, –CF_3_ and –CH_3_ groups	synthesis within the work	-	Flat-sheet	Gas separation	[13]
–SO_3_H groups	Sigma-Aldrich, St. Louis, MO, USA	M_n_ ~22	Flat-sheet	Polymer fuel cells	[14]
*N*-dodecyl-4(*R*)-hydroxy-l-proline	BASF, Ludwigshafen, Germany	-	Flat-sheet	Dialysis	[15]
phosphonic acid (HO)_2_(O)PH_2_C–	Sigma-Aldrich, St. Louis, MO, USA	M_w_ ~35	Flat-sheet	Ion exchange membrane	[16]
2-ethyl-2-oxazoline	synthesis within the work	M_n_ ~15.2	Flat-sheet	Water purification	[17]
polymethacrylates	Solvay Advanced Polymers, Düsseldorf, Germany	M_n_ ~29	Flat-sheet	Boron removal	[18]
CF_3_-groups, poly(ethylene glycol)	synthesis within the work	M_n_ ~5.2 M_w_ ~28.6	Flat-sheet	CO_2_/CH_4_ separation	[19]
poly(tertbutylacrylate)	Sigma-Aldrich, St. Louis, MO, USA	M_n_ ~22	Flat-sheet	UF	[20]
poly(N,N-dimethylamino-2-ethylmethacrylate)	Solvay Advanced Polymers	M_w_ ~17.6	Flat-sheet	Hemodialysis	[21]

**Table 2:** Synthesized PSF samples and synthesis conditions.

PSF	Solvent	Molar Ratio DCDPS: Bisphenol A
1	DMAc	1:1.01
2		1:1.015
3		1:1.025
4		1.01:1
5		1.018:1
6		1.03:1
7		1:1
8	NMP	1:1
9	DMSO	1:1

**Table 3:** Molecular weight characteristics of synthesized PSF determined by the GPC method and by estimates made based on the NMR results.

PSF	(-OH):(-Cl)	M_w_, kg/mol	M_n_, kg/mol	M_w_/M_n_	Estimated Number of Links	M_NMR_, kg/mol
1	2.6:1	65	27	2.4	56	28
2	2.7:1	58	20	2.9	50	25
3	6.9:1	57	12	4.8	41	20
4	1:1.6	128	34	3.8	60	30
5	1:2.7	43	18	2.4	49	24
6	1:3.4	37	12	3.1	39	20
7	1.2:1	55	28	2.0	79	39
8	1.3:1	79	33	2.4	92	45
9	1.2:1	22	4	5.8	10	5

**Table 4:** The coagulation rate of PSF/NMP/PEG-400 dope solutions (21/49/30 wt.%).

Sample	η, Pa·s	υ, µm/s
PSF-1	32	4.9
PSF-7	15	6.0
PSF-8	56	3.9

**Table 5:** Properties of the hollow fiber membrane obtained in the work.

Sample	D_out_, mm	δ, µm	*P/l* (N_2_), (m^3^/bar·m^2^·h)	*P/l* (He), (m^3^/bar·m^2^·h)	α (He/N_2_)	d_max_, nm	d_MFP_, nm
PSF-1	0.8	150	20	45	2.3	28	23
PSF-7	0.9	200	45	73	1.6	81	27
PSF-8	0.8	100	1.2	2.3	1.9	35	18

## Data Availability

Not applicable.
